# Postnatal Anthropometric and Body Composition Profiles in Infants with Intrauterine Growth Restriction Identified by Prenatal Doppler

**DOI:** 10.1371/journal.pone.0150152

**Published:** 2016-03-03

**Authors:** E. Mazarico, R. Martinez-Cumplido, M. Díaz, G. Sebastiani, L. Ibáñez, M. D. Gómez-Roig

**Affiliations:** 1 BCN-Barcelona Center of Maternal-Fetal Medicine and Neonatology (Hospital Sant Joan de Déu and Hospital Clínic), Fetal i+D Fetal Medicine Research Center, Barcelona, Spain; 2 Spanish Maternal and Child Health Network, Retic SAMID, Institute Carlos III, Madrid, Spain; 3 IDIBAPS, University of Barcelona, Barcelona, Spain; 4 Centre for Biomedical Research on Rare Diseases (CIBER-ER), Barcelona, Spain; 5 Endocrinology Unit, Sant Joan de Déu University Hospital, Barcelona, Spain; 6 Centre for Biomedical Research on Diabetes and Associated Metabolic Diseases (CIBERDEM), Madrid, Spain; Hôpital Robert Debré, FRANCE

## Abstract

**Introduction:**

Infant anthropometry and body composition have been previously assessed to gauge the impact of intrauterine growth restriction (IUGR) at birth, but the interplay between prenatal Doppler measurements and postnatal development has not been studied in this setting. The present investigation was performed to assess the significance of prenatal Doppler findings relative to postnatal anthropometrics and body composition in IUGR newborns over the first 12 months of life.

**Patients and Methods:**

Consecutive cases of singleton pregnancies with suspected IUGR were prospectively enrolled over 12 months. Fetal biometry and prenatal Doppler ultrasound examinations were performed. Body composition was assessed by absorptiometry at ages 10 days, and at 4 and12 months.

**Results:**

A total of 48 pregnancies qualifying as IUGR were studied. Doppler parameters were normal in 26 pregnancies. The remaining 22 deviated from normal, marked by an Umbilical Artery Pulsatility Index (UA-PI) >95^th^ centil or Cerebro-placental ratio (CPR) <5^th^ centile. No significant differences emerged when comparing anthropometry and body composition at each time point, in relation to Doppler findings. Specifically, those IUGR newborns with and without abnormal Doppler findings had similar weight, length, body mass index, lean and fat mass, and bone mineral content throughout the first 12 months of life. In a separate analysis, when comparing IUGR newborns by Doppler (abnormal UA-PI vs. abnormal CPR), anthropometry and body composition did not differ significantly.

**Conclusions:**

Infants with IUGR maintain a pattern of body composition during the first year of life that is independent of prenatal Doppler findings. Future studies with larger sample sizes and correlating with hormonal status are warranted to further extend the phenotypic characterization of the various conditions now classified under the common label of IUGR.

## Introduction

Normal fetal growth results from a genetically pre-determined growth potential, which in turn is modulated by maternal, fetal, placental, and environmental factors [1]. In case of intrauterine growth restriction (IUGR), this genetically endowed growth potential fails to materialize. IUGR is normally used to refer to small fetuses with higher risk for fetal in utero deterioration, stillbirth and overall poorer perinatal outcome as compared with normally grown fetuses. In general, IUGR is associated with Doppler signs suggesting hemodynamic redistribution as reflection of fetal adaptation to undernutrition/hypoxia, histological and biochemical signs of placental disease and higher risk of preeclampsia. On the other hand, constitutionally small fetuses (SGA) typically weigh less, and placental insufficiency at term may not be evident in umbilical arterial Doppler studies, so that a diagnosis of IUGR often represents a challenge. The term SGA has been used to differentiate a sub-group of small fetuses that do not present the changes above described, so that there appear to be no fetal adaptation to an abnormal environment, and with perinatal outcomes similar to those of normally grown fetuses. Still, the importance of differentiating these conditions cannot be overstated. As IUGR is a major contributor to stillbirths and perinatal morbidity, small for gestational age (SGA) births merely represent the low end of normal infant size distribution [2].

As yet, no single parameter has proven pivotal in distinguishing between late-onset IUGR and SGA status. However, abnormal Doppler studies (i.e., uterine arterial indices and cerebroplacental ratio [CPR]) signaling placental compromise and severe growth restriction (<3^rd^ centile) have been identified as risk factors for adverse outcomes in SGA infants, implying that such births instead qualify as IUGR [3,4].

A number of short- and long-term health risks have also been identified with respect to SGA infants [5]. It is generally acknowledged that extremes of normal birth weight heighten perinatal morbidity and mortality risk. In addition, IUGR has long-term metabolic, neurologic, and cardiovascular consequences, as a result of the so-called fetal programming [6–12]. Infant anthropometrics and body composition have been evaluated in various studies to gauge the impact of IUGR at birth [13–17], but the interplay between Doppler measurements and postnatal development has not been studied in this setting.

The present study aimed at assessing the significance of Doppler findings in relation to postnatal anthropometrics and body composition in IUGR newborns throughout the first 12 months of life.

## Material and Methods

### Subjects

Consecutive singleton pregnancies with suspected IUGR, spanding over a 12-month period at our outpatient Obstetrics clinic, were prospectively recruited for the study. IUGR was suspected if estimated fetal weight was below 3^rd^ centile with normal or abnormal uterine arterial Doppler or cerebroplacental ratio [18].

IUGR was confirmed after birth in all newborns fulfilling the following criteria: 1) birth weight <3rd centile with normal Doppler; or 2) birth weight <3^rd^ centile with abnormal uterine arterial Doppler study (pulsatility index [PI] >95^th^ centile) [19]; or 3) birth weight <3^rd^ centile with abnormal cerebroplacental ratio (CPR <5^th^ centile) [20]. Exclusion criteria were: 1) gestational age <37 weeks at delivery; 2) maternal use of drugs that may affect fetal growth or biochemical markers (including steroids); 3) gestational hypertension; and 4) preeclampsia [21].

All patients included were normotensive and all deliveries were dated by crown-rump lengths measured in the first trimester [22].

### Doppler measurements

In each case, fetal biometry and prenatal Doppler ultrasound (US) examinations were performed by experienced operators using either a Siemens Sonoline Antares (Siemens Medical Systems, Malvern, PA, USA) or a General Electric Voluson E8 (GE Medical Systems, Zipf, Austria) unit equipped with a 6–2 MHz linear curved-array transducer. Estimated fetal weight (EFW) was calculated from biparietal diameter, head and abdominal circumferences, and femoral length using the Hadlock formula [23]. The PI of the umbilical artery (UA) was calculated from a free-floating portion of the umbilical cord. To minimize variability, the PI of middle cerebral artery (MCA) was also measured (in the transverse view of fetal head) at its origin from the circle of Willis [19]. Cerebroplacental ratio (CPR) was then calculated as MCA-PI divided by UA-PI [20]. To assess uterine artery, a US probe was placed on the lower abdominal quadrant, angled medially; and color Doppler imaging was again engaged to identify the UA at its apparent intersection with the external iliac artery. Measurements were taken approximately 1 cm distal to the perceived junction. Doppler recordings were performed in the absence of fetal movements and with voluntary maternal suspension of respiration. All pulsed Doppler indices were generated automatically from at least three consecutive waveforms, with the angle of insonation as close to 0 as possible and always less than 30°. A high-pass wall filter of 70 Hz was used to record low flow velocities and to avoid artifacts. The last Doppler evaluation within 1 week of delivery was referenced for data analysis.

### Body composition

Body composition was assessed by absorptiometry at 10 days of age, and at 4 and 12 months using a Lunar Prodigy bundled with proprietary software (v3.4/3.5; Lunar Corp, Madison, WI, USA) adapted for infants [13,14]. All body composition studies were performed during spontaneous sleep prior to feeding. Body fat, abdominal fat, lean mass, and bone mineral content (BMC) were assessed. Coefficients of variation (CVs) were 3% for fat and lean mass [13,14].

### Statistics and ethics

All data were expressed as mean ± standard error of the mean. Student’s *t*-test was applied for quantitative variables of normally distributed data. Non-normally distributed data were compared via non-parametric Mann-Whitney U test (two categories). Standard software (SPSS v19.0 for PC; SPSS Inc, Chicago, IL, USA) was utilized for all calculations, setting statistical significance at *p*<0.05.

The study protocol was approved by the Institutional Review Board of Barcelona University Hospital. Written informed consent was granted from the parents or guardians of all participants.

## Results

A total of 48 pregnancies qualifying as IUGR were studied. Doppler parameters were normal in 26 pregnancies. The remaining 22 deviated from normal, marked by al UA-PI >95^th^ centile [19] or CPR <5^th^ centile [20].

Anthropometry and body composition profiles were adjusted for gestational age and gender.

At the time of delivery, IUGR births with normal and abnormal Doppler findings differed significantly in terms of gestational age and weight percentile, although all of them were below <3rd centile. There were no significant differences in birth weight, birth length or BMI (Table 1).

**Table 1:** Anthrompometry and body composition of IUGR with normal and abnormal Doppler.

	IUGR with normal Doppler				IUGR with abnormal Doppler			
	Birth	10 days	4 months	*12 months*	Birth	10 days	4 months	*12 months*
N	26	26	24	*10*	22	22	14	*11*
Girls (%)	42	42	45	*43*	48	48	45	*48*
***Auxology***^¶^ Gestational age (wk)	38.2 ± 0.2	-			37.7 ± 0.2 ^a^	-		
Birth Weight (g)	2279 ± 290	-			2208 ± 210	-		
Birth Weight (percentile)	0.85 ± 0.2	-			1.6 ± 0.4^a^	-		
Birth Length (cm)	45.5 ± 1.1	-			45.2 ± 1.3	-		
BMI (Kg/m^2^)	11.0 ± 2.4	-			10.8 ± 1.2	-		
***Endocrinology*** IGF-I (ng/mL)^§^	48.43 ± 9.67	-	68.52 ± 5.37	70.52 ± 8.27	40.62 ± 3.61	-	65.72 ± 5.33	56.13 ±10.01
***Body Composition***^#^ Length(cm)		47.2 ± 1.0	60.1 ± 1.8	72.7 ± 6.0		44.7.5 ± 1.2	60.0 ± 2.3	73.5 ± 4.1
Weight (g)		2704 ± 177	6029 ± 797	8818 ± 1710		2629 ± 234	5778 ± 837	8710 ± 1070
BMI (Kg/m^2^)		12.1 ± 1.4	16.6 ± 1.4	16.5 ± 2.0		11.6 ± 1.4	16.0 ± 1.1	16.1 ± 1.7
Fat Mass (g)		471 ± 283	1880 ± 254	3403 ± 853		408 ± 213	1902 ± 172	3520 ± 717
Lean Mass (g)		2240 ± 774	3870 ± 544	5451 ± 1137		2267 ± 620	3730 ± 678	5359 ± 700
Bone mineral density (g/cm^2^)		0.19 ± 0.06	0.30 ± 0.03	0.35 ± 0.14		0.18 ± 0.05	0.32 ± 0.05	0.36 ± 0.05

Values are mean ± SEM. IUGR, intrauterine growth restriction; BMI, body mass index

^§^ IGF-I was assessed at birth in cord blood, and at 4, 12 & 24 mo in peripheral blood in fasting state

^¶^ Anthropometric values (mean ± SEM) in newborns with gestational age 38 wk: Boys, birth weight, 3175 ± 17; birth length, 49.1 ± 0.1; Girls, birth weight, 3017 ± 16; birth length, 48.9 ± 0.1 (Ferrández et al., 2004)

^**#**^ Normative values in appropriate-for-gestational-age infants [birth weight between -1SD and +1SD (approximately percentiles 25–75) according to the Spanish growth charts] are shown in Table in S1 Table.

^a^ p<0.05 adjusted for gender

No significant differences emerged either when comparing anthropometry and body composition at age 10 days, and at 4 and 12 months according to Doppler findings (Table 1 and Table in S1 Table). Specifically, IUGR births with and without abnormal Doppler were similar in weight, length, BMI, lean mass, fat mass, and BMC during the 12-month follow-up period.

In a separate analysis, comparing IUGR births by Doppler findings (abnormal UA-PI vs. abnormal CPR), anthropometry and body composition did not differ significantly.

IGF-1 concentrations either in cord blood or in the first year of life were not related to the presence or absence of Doppler abnormalities (Table 1).

## Discussion

Birth weight reflects fetal growth in utero and is determined by a number of variables, such as gender, maternal height/weight, parity, and ethnicity. IUGR signifies that anticipated fetal growth has fallen short [1]. Differentiating SGA infants, marked by constitutional smallness, from IUGR births often presents a diagnostic challenge. However, it is important to do so, given the increased risk of perinatal morbidity and mortality attached to IUGR, as well as the long-term metabolic, cardiovascular, and neurologic consequences of fetal programming *in utero* [24–31].

Usually, IUGR status is associated with Doppler imaging evidence of hemodynamic redistribution (stemming from fetal adaptation to undernutrition/hypoxia), in addition to histologic and biochemical evidence of placental disease [1]. Thus, Doppler indices, such us UA-PI and CPR, may help to identify the settings (as above) where worse perinatal and long-term outcomes are anticipated [3, 4].

Several previous studies have already investigated the evolving body composition in infants with low birth weights, describing a characteristic pattern (less lean mass, fat mass, and bone mineral density) bearing higher metabolic risk [13,14,32,33]. However, they failed to distinguish the presence of IUGR from low birth weight with no signs of placental insufficiency or hypoxia, based on Doppler findings.

This study is the first to assess postnatal body composition in infants with and without abnormal Doppler determinants. Our results are aligned with those in previous reports, showing less lean mass, fat mass, and bone mineral density relative to infants of normal birth weight. However, one might expect an eventual recovery of normal fetal growth and body composition in IUGR due to placental insufficiency, once hypoxia and undernutrition subside. However, we found no differences in body composition during the first 12 months of postnatal life, regardless of whether placental insufficiency, shown by Doppler abnormalities, was present or not. Accordingly, it seems that birth weight percentile determines body composition over the first few years in case of IUGR, independently of the etiology.

The main strengths of the study are its longitudinal nature and the assessment of body composition by absorptiometry in newborns and very young infants. Among the study limitations were the relatively small sampling size, the loss of follow-up in a percentage of the study population, the lack of hormonal assessments, and the non-inclusion of other subsets of patients, such as SGA and appropriate-for-gestational-age newborns. In conclusion, we disclose that infants with IUGR maintain a uniform pattern of body composition during the first year of life, independent of Doppler findings during pregnancy. Additional studies in larger populations, including hormonal assessments, are warranted to further delineate phenotypically assorted conditions presently bearing the common label of IUGR.

## Supporting Information

S1 Table Body composition of infants born appropriate-for-gestational age (AGA, n = 31) exclusively breast-fed in early infancy (0–4 mo). (DOCX)
