# Correction: Postnatal Anthropometric and Body Composition Profiles in Infants with Intrauterine Growth Restriction Identified by Prenatal Doppler

**DOI:** 10.1371/journal.pone.0157194

**Published:** 2016-06-03

**Authors:** E. Mazarico, R. Martinez-Cumplido, M. Díaz, G. Sebastiani, L. Ibáñez, M. D. Gómez-Roig

## Notice of Republication

The original S1 Dataset was published in error. The S1 Dataset was removed from the XML and PDF versions of this article on May 18, 2016.
