# Supplementary material for: Postnatal Anthropometric and Body Composition Profiles in Infants with Intrauterine Growth Restriction Identified by Prenatal Doppler
Source: PLoS One. 2016 Mar 3;11(3):e0150152. doi: 10.1371/journal.pone.0150152 (PMC4777361; doi:10.1371/journal.pone.0150152)
Supplement: S1 Table — (DOCX) [file pone.0150152.s001.docx]

**S1 Table**

Body composition of infants born appropriate-for-gestational age (AGA, n=31)

exclusively breast-fed in early infancy (0-4 mo).

|  | **At 2 wk** | **At 4 mo** | **At 12 mo** |
| --- | --- | --- | --- |
| Girls (n,%) | 17 (52) | - | - |
| Gestational age (wk) | 39.8 ± 0.1 | - | - |
| Birth Weight (kg) | 3.4 ± 0.1 | - | - |
| Birth Length (cm) | 49.8 ± 0.2 | - | - |
| Fat Mass (kg) | 0.8 ± 0.1 | 2.7 ± 0.1 | 3.8 ± 0.1 |
| Lean Mass (kg) | 3.0 ± 0.1 | 4.2 ± 0.1 | 6.2 ± 0.2 |
| BMD (g/cm^2^) | 0.24 ± 0.01 | 0.29 ± 0.01 | 0.39 ± 0.01 |

Values are mean ± SEM.

^a^ p<0.05, ^b^ p<0.01, ^c^ p<0.001 and ^d^ p<0.0001 vs AGA-BRF.
